# On Rheumatism, Gout, and Sciataca

**Published:** 1864

**Authors:** 


ON RHEUMATISM, GOUT, AND SCIATACA. Their Pathology, Symptoms, and Treatment. By HENRY WILLIAM FULLER, M.D., Contab., &c., &c. Third edition. Philadelphia: LINDSAY & BLAKISTON. 1864.

FULLER'S work on Rheumatism has, in a great measure, revolutionized the practice of the profession in that disease. The present prominence accorded to the alkaline treatment is due, to a considerable extent, to the influence of past editions of this work.

It is not necessary to dilate at present on the great merits of a work so widely known. Suffice it to say that no medical library is reasonably complete without it. The present edition is more full than the former ones on the subjects of Chronic Rheumatism, Neuralgic Rheumatism, Sciatica, and Rheumatic Gout. The following are the topics discussed:—

Chapter I. The Rheumatic Poison.
" II. " Rheumatic Diathesis.
" III. " Location and Classification of Rheumatism.
" IV. & V. Of Acute Rheumatism.
" VI. VII. VIII. & IX. Rheumatism of the Heart.
" X. Of Rheumatic Affection of the Brain and other Organs.
" XI. Of Rheumatic Gout.
" XII. Of Chronic Rheumatism.
" XIII. Of Sciatica and other Forms of Neuralgic Rheumatism.

